# pH of anti-VEGF agents in the human vitreous: low impact of very different formulations

**DOI:** 10.1186/s40942-017-0075-x

**Published:** 2017-06-26

**Authors:** Bianka Sobolewska, Peter Heiduschka, Karl-Ulrich Bartz-Schmidt, Focke Ziemssen

**Affiliations:** 1 Center for Ophthalmology, Eberhard-Karl University Tübingen, Elfriede-Aulhorn-Straße 7, 72076 Tübingen, Germany; 2 Department of Ophthalmology, University of Münster Medical Center, Münster, Germany

**Keywords:** Bevacizumab, Ranibizumab, Aflibercept, Ziv-aflibercept, Rituximab, Anti-VEGF, Vitreous, pH

## Abstract

**Background:**

The aim of the study was to measure pH changes of the human vitreous caused by the intravitreal drugs bevacizumab, ranibizumab, aflibercept, and ziv-aflibercept.

**Methods:**

Fresh human vitreous samples were obtained during core vitrectomy (23-gauge) from patients with epiretinal gliosis. Aliquots of bevacizumab, ranibizumab, aflibercept or ziv-aflibercept (2 µl) were added consecutively to 200 µl of vitreous samples or 0.9% NaCl saline. The pH was measured using a pH-sensitive microelectrode. Rituximab, in off-label use against intraocular lymphoma, was tested as an IgG1 antibody.

**Results:**

The pH of the administered drugs was 5.91 for bevacizumab (95% CI 5.63–6.19), 5.32 for ranibizumab (95% CI 5.0–5.63), 6.05 for aflibercept (95% CI 5.78–6.31), ziv-aflibercept 6.1 (95% CI 6.05–6.15), and 6.29 for rituximab (95% CI 5.97–6.61). While the fresh and undiluted vitreous fluid showed pH values of 7.0–7.4, pH values increased if saline or rituximab were added. In contrast, the pH decreased slightly if aflibercept, bevacizumab, ranibizumab or ziv-aflibercept were supplemented. The observed pH decreases were not significant after ranibizumab was added. Significant changes were only notable with higher-than-normal amounts of bevacizumab (26–40 µl). The vitreous showed the most robust buffering capacity towards ranibizumab and rituximab.

**Conclusions:**

The pH changes in vitreous samples elicited by the usual intravitreal anti-VEGF drugs differed clearly, but only by much higher concentrations than used in the clinical routine. Although the ingredient solution of ranibizumab showed the lowest pH, it caused only moderate changes of vitreal pH compared to bevacizumab, aflibercept or ziv-aflibercept.

## Background

The anti-vascular endothelial growth factor (anti-VEGF) agents are the first-line treatment in neovascular age-related macular degeneration (AMD) and other frequent ocular diseases associated with macular edema and retinal neovascularization. Of the three different anti-VEGF agents available, ranibizumab (recombinant F_ab_ antibody fragment) and aflibercept (fusion protein), have been approved by the Food and Drug Administration for intravitreal application. Bevacizumab (IgG1 antibody) and ziv-aflibercept (identical fusion-protein to aflibercept) are used off-label because they were primarily developed for intravenous treatment of metastatic colorectal cancer [1, 2].

All of these anti-VEGF agents differ in their clinical formulation. Since a different pH and components of drug solutions may lead to changes in the buffering capacity of the human vitreous, the purpose of this study was to evaluate the pH changes of the human vitreous after the administration of intravitreous drugs. The effects of rituximab, used for treatment of intraocular lymphoma, were also characterized [3].

## Methods

### Materials

Bevacizumab was obtained from Roche Pharma (Basel, Switzerland), ranibizumab from Novartis Pharma GmbH (Nürnberg, Germany), aflibercept from Bayer Pharma (Berlin, Germany), and ziv-aflibercept from Sanofi-Aventis (Frankfurt, Germany). The CD20-specific humanized monoclonal antibody rituximab, which served as an antibody control, was obtained from Roche Pharma (Basel, Switzerland).

### Sample collection

Human vitreous samples were collected by 23-gauge pars-plana core vitrectomy for epiretinal gliosis at a tertiary center (Center for Ophthalmology at the University of Tübingen, Germany). All subjects (n = 10, women: men 4:6, age: mean = 70.5 years (95% CI 67.3–73.6), phakic: pseudophakic eyes 5:5, emmetropic:myopic eyes 7:3, a spherical equivalent was below 3 diopters) with any signs of pre- or intraoperative hemorrhage, any history of previous vitreoretinal surgery or intravitreal drug injections or any additional vitreoretinal disease were excluded from the study. The study adhered to the tenets of the Declaration of Helsinki and the Institutional Ethics Committee of the University of Tübingen granted approval. Written informed consent was obtained from all patients.

### pH measurements

A high-quality, micro-pH electrode (HI 108213 HANNA^®^ Instruments France PHR-146S) was used to determine pH values in small volumes. The recording system was calibrated before each experiment. The values of the drugs were determined in ready-to-use vials and syringes. For the fresh vitreous samples, all measurements were performed at least three times in ambient air and at room temperature, immediately after the collection. Up to 40 µl of bevacizumab, ranibizumab, aflibercept, ziv-aflibercept or rituximab was added in consecutive 2-µl steps to 200 µl of homogenized vitreous sample or 0.9% NaCl (Gibco^®^, USA, pH 7.0–7.3) [4]. If *we* assume the vitreous volume to be 4.4–5.5 ml (50 µl = 1/88–1/110), the 2 µl might correspond very likely to the effect of a single injection (1/100).

The pH of the gently mixed sample was measured continuously, and values were recorded after stabilization of displayed pH after each addition [4].

### Statistics

Measured data were evaluated by non-parametric routines using the GraphPad Prism 6.0 software. The significance of differences was tested by the Friedman test (analysis of matched data sets) with the Dunn’s multiple comparisons test, where baseline data (pH values before addition of drugs) were compared with data after the addition of each drug. Correlations given in Fig. 2 were checked by Spearman’s test (pairwise x–y comparison).

## Results

The pH values of the undiluted agents were measured, and the corresponding formulations and pH values are listed in Table 1. The ingredient solution containing ranibizumab showed the lowest pH of all three anti-VEGF agents (pH 5.32, 95% CI 5.00–5.63). Bevacizumab, aflibercept, and ziv-aflibercept had similar pH values of 5.91 (95% CI 5.63–6.19), 6.05 (95% CI 5.78–6.31) and 6.1 (95% CI 6.05–6.15), respectively, even though they are composed of different components.

**Table 1:** The formulations and pH values of anti-VEGF agents

Drug	Concen-tration (osmola-rity)	Dose in the vitreous (4 ml)/dose in 0.002 ml	Formulation	Measured pH	95% CI
Ranibizumab (Lucentis)	20 mg/ml (289 mOsm)	0.5 mg/0.04 mg	10 mM histidine-HCl, 10% α,α-trehalose dihydrate, 0.01% polysorbate 20	5.32	5.0–5.63
Bevacizumab (Avastin)	25 mg/ml (182 mOsm)	1,25 mg/0.05 mg	42 mM NaH_2_PO_4_·H_2_O, 8.45 mM Na_2_HPO_4_, 6% α,α-trehalose dihydrate, 0.04% polysorbate 20	5.91	5.63–6.19
Aflibercept (Eylea)	40 mg/ml (1000 mOsm)	2 mg/0.08 mg	10 mM Na_3_PO_4_, 40 mM NaCl, 5% sucrose, 0.03% polysorbate 20	6.05	5.78–6.31
Ziv-aflibercept (Zaltrap)	25 mg/ml (1000 mOsm)	1.25 mg/ 0.05 mg	100 mM NaCl, 5 mM Na citrate, 5 mM Na_3_PO_4_, 20% sucrose, 0.1% polysorbate 20	6.1	6.05–6.15
Rituximab (Rituxan)	10 mg/ml	1 mg/0.02 mg	154 mM NaCl, 25 mM Na citrate·2H_2_O, 0.07% polysorbate 80	6.29	5.97–6.61

**Fig. 1:**
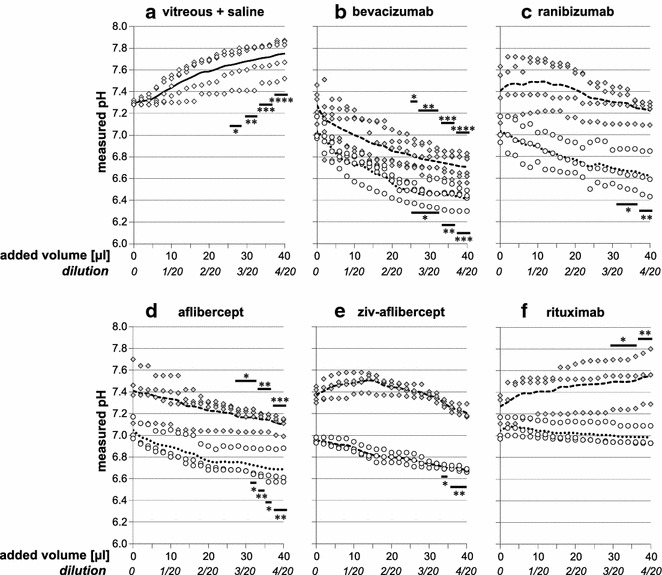
**a** pH changes measured after addition of saline to vitreous. **b**–**e** pH changes measured after addition of various drugs as indicated to vitreous (*diamonds*) or to saline (*circles*). *Broken lines* (addition to vitreous) or *dotted lines* (addition to saline) indicate mean values. Significance of differences to initial values was calculated by Dunn’s multiple comparison and indicated by asterisks with *p < 0.05, **p < 0.01, ***p < 0.001 and ****p < 0.0001

The mean pH of the fresh vitreous samples before addition of any drug was 7.29. In the absence of any visible inclusions such as blood cells, there was some variability leading to a confidence interval (95% CI) of 7.20–7.39. The influence of the administered drugs on the measured pH values was evaluated in titration experiments and is shown in Fig. 1. When only saline was added to the vitreous, the pH increased by 0.46 units (Fig. 1a). After the addition of drugs to the saline, the pH decreased to different extents (open circles in Fig. 1b–e). The decrease was the smallest and not significant when rituximab was added (0.06 pH units compared to 0.62, 0.41, 0.36 and 0.27 units for bevacizumab, ranibizumab, aflibercept, and ziv-aflibercept, respectively). However, the pH values varied significantly when the drugs were added to vitreous (grey diamonds in Fig. 1b–e).

Whereas the pH increased by 0.28 units after the addition of rituximab to vitreous (Fig. 1e), the pH decreased clearly and to almost the same extent as in saline after the addition of aflibercept (0.31 units) and more clearly with bevacizumab (0.57 units). When ranibizumab and ziv-aflibercept were added, the pH increased slightly at first and then decreased slightly, resulting in a final change of 0.18 units and 0.2 units, respectively.

**Fig. 2:**
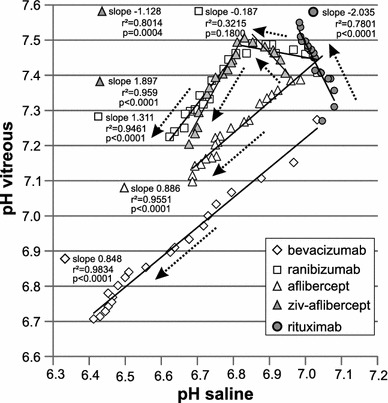
Diagram showing correlation between changes of pH in saline and vitreous. Orientation of the *arrows* shows the course of increasing amount of added drugs. Slopes and correlation coefficients were calculated by linear regression, and p values by Spearman correlation

In order to compare pH changes in vitreous and in saline, mean pH values after the addition of the same volumes were plotted in a correlation diagram (Fig. 2). The arrows near the data points show the direction of pH changes during titration. There was an almost perfect linear correlation between pH changes in saline and vitreous when aflibercept or bevacizumab were added, and the pH decreased slightly less in vitreous than in saline. However, a two-phasic trend was observed when ranibizumab or ziv-aflibercept was added. Firstly, the vitreous pH remained almost constant, whereas the saline pH decreased after addition of the same amount of these drugs. The pH decreased after the addition of more than 16 µl of both ranibizumab and ziv-aflibercept. In contrast, the addition of rituximab to vitreous resulted in a pH change in the opposite direction as that resulting from the addition of rituximab to saline.

## Discussion

For intravitreal drugs, the data showed differences between the individual formulations and buffering systems. In spite of some fluctuations, however, the buffering capacity of the human vitreous fluid substantially reduced the effects of the drugs on the intraocular pH. Therefore, the pH changes induced by ranibizumab, rituximab and ziv-aflibercept (with slight restrictions) also by aflibercept and bevacizumab occurred in a small range and thus were found to be harmless. It would be fallacious to speculate on the potential detrimental effects of the undiluted agents on single cells or in different environments (e.g., after subretinal injection).

The concept of pH was developed by the Danish biochemist, Søren Peter Lauritz Sørensen in 1909 [5] using the negative form of the logarithm to base 10, in which p represented the numerical value of the power of the hydrogen ion exponent. The ‘titratable acidity’ can be measured by titration to pH 7.4 with sodium hydroxide. Although the buffering capacity of saline is negligible, buffering limits the effect of adding free acid of low titratable acidity, what is less easily accommodated. Weak anions, such as bicarbonate or albumin can associate with or liberate protons according to the prevailing [H^+^], effectively ‘buffering’ the pH around the dissociation constant of that acid anion. Administration of a 0.9% saline solution, containing no buffer base, was shown to dilute the endogenous buffer systems in the plasma, such as the important CO_2_/HCO_3_- buffer system [6]. The vitreous body is a highly hydrated gelatinous structure that consists of collagenous and non-collagenous glycoproteins, glycosamino-glycans, hyalocytes and fibroblasts [7, 8]. Its biochemical composition and anatomy have been extensively investigated [9], but still show interindividual differences [10]. However, not all physiological functions of the vitreous body are well understood [8, 9]. Until now, there were only a few reports about pH changes in the vitreous body [4, 11, 12]. Further knowledge of the buffering capacity of the vitreous should help in the development of well-tolerated, sustained drug-delivery systems and vitreous substitutes.

In this study, we evaluated the pH changes of the human vitreous after the addition of four anti-VEGF agents. Bevacizumab, ranibizumab, and aflibercept account for the vast majority of intravitreal injections in posterior segment diseases. In order to examine the buffering capacity of the human vitreous, these agents were also added to saline for comparison. Moreover, to check the pH drift of the vitreous in ambient air, saline was added to vitreous in another control experiment: If only neutral saline was added to the vitreous, the pH drifted toward the basic direction (Fig. 1a), as also reported by Conway et al. [4] It was therefore of interest to determine whether the addition of slightly acidic solutions of antibodies to the vitreous would reduce this basic shift or even result in a pH shift toward an acidic direction.

The vitreous was able to buffer acidic antibody solutions, yielding a slower decrease of pH than in saline or even a stable pH for at least a while. In detail, the results showed that the vitreous has a smaller buffering capacity towards aflibercept and bevacizumab solutions. The pH decreased when both drugs were added, though to a slightly smaller extent than after the addition of aflibercept or bevacizumab to saline (indicated by the slopes smaller than 1 in Fig. 2). In contrast, the pH remained almost stable after the addition of a small amount of both ranibizumab and ziv-aflibercept solution (16 µl, dilution: 1:12.5), thereafter the pH began to shift in an acidic direction. This result can be explained by the weak buffering capacity of histidine-HCl and sodium citrate, respectively (Table 1). Although ziv-aflibercept contains the same active drug as aflibercept, there are some differences in formulations of aflibercept and ziv-aflibercept, which seem to explain the above depicted ph changes in the human vitreous after addition of these drugs. However, only slight ph changes in the human vitreous after addition of ziv-aflibercept was observed. Therefore, this results corroborates previous data on safety use of ziv-aflibercept [2, 13].

The pH of rituximab (6,5) was close to neutral. Indeed, its addition to vitreous resulted in a slight increase rather than a decrease of pH, although to a lesser extent than if only saline was added. Moreover, the decline in pH of saline was much slower after rituximab was added than after addition of any of the anti-VEGF drugs.

The slight ability of the vitreous to resist changes in pH after the addition of acidic or alkaline drugs can be explained by its bicarbonate buffer system, an important intrinsic buffering capacity of the vitreous. Conway et al. showed that the buffering capacity of bovine vitreous to 0.1 N HCl and 0.1 N NaOH was greater than that of 0.9% NaCl, which is not surprising because saline does not have any buffering capabilities at all [5].

The bevacizumab solution caused the most pronounced pH shift in the acidic direction of both saline and vitreous (Fig. 1b). The complete buffer system present (Na_2_HPO_4_/NaH_2_PO_4_) in the commercially available formulation of bevacizumab may prevent the pH of bevacizumab to be changed and thus counteract the vitreous buffer system. Components of the other antibody solutions also have certain buffering capabilities; however, by far not as strong as the Na_2_HPO_4_/NaH_2_PO_4_ couple used in the bevacizumab solution.

Several limitations of this study have to be considered: (1) An open system that could lead to a slight alkaline pH drift [12, 14]. (2) Lack of measurement of the hyaluronate acid concentration in the vitreous samples, which might additionally act as a buffer, and (3) “gently mix” of homogenized vitreous samples. Furthermore, the extracted vitreous might show reactions other than those occurring in the living eye, including a lack of diffusion of substances into the vitreous across the blood-retinal barrier [11]. Moreover, it is likely that there are no living hyalocytes and fibroblasts left in the vitreous samples used in our experiments. These cells may also contribute to pH stabilization in the living eye. On the other hand, it has to be pointed out that even intravitreal pH measurements are invasive and not free of artifacts [12].

The higher concentrations of anti-VEGF drugs in our experiments do definitely exceed the actual levels typical of clinical use. Therefore, in vitro experiments cannot be directly applicable to the situation in the living eye. Further studies with a large number of patients are warranted to clarify the impact of those factors on pH changes of the human vitreous and set up correlations between refraction or lens status and buffering capacity of the human vitreous.

In conclusion, the vitreous maintains a stable pH after the addition of small amounts of anti-VEGF drugs, which is of relevance for clinical use [15]. Although the human vitreous has a certain buffering capacity, it is not able to maintain an acid-base balance after the addition of higher-than-normal amounts of bevacizumab and aflibercept in our *ex vivo* study. In order to assure the same buffering capacity of the in vitro experiments, media with a buffering capacity similar to that of the vitreous should be developed. As long as media with different contents (e.g., fetal bovine serum) are used in cell culture, non-physiological pH conditions have to be taken into consideration when in vitro results are evaluated [16]. The pH of drugs might affect sensitive cells. The direct contact with undiluted agents should be carefully tested before used in the sub-retinal or intra-retinal compartment. Additional research is required to examine the effects of drug application under physiologic conditions (i.e., under 5 vol% CO_2_ and temperature of 33°C [14]). Moreover, the impact of temperature differences on the human vitreous buffering capacity is worthy of examination [12, 14].

## Conclusions

The experiments demonstrated that the buffering capacity of the human vitreous is an important factor in preventing harmful pH variations, which might otherwise be very likely facing the strongly acidic formulations of some intravitreal agents.

## References

[1] Ziemssen F, Sobolewska B, Deissler H, Deissler H (2016). Safety of monoclonal antibodies and related therapeutic proteins for the treatment of neovascular macular degeneration: addressing outstanding issues. Expert Opin Drug Saf.

[2] de Oliveira Dias JR, de Andrade GC, Novais EA, Farah ME, Rodrigues EB (2016). Fusion proteins for treatment of retinal diseases: aflibercept, ziv-aflibercept, and conbercept. Int J Retina Vitreous.

[3] Larkin KL, Saboo US, Comer GM, Forooghian F, Mackensen F, Merrill P, Sen HN, Singh A, Essex RW, Lake S, Lim LL, Vasconcelos-Santos DV, Foster CS, Wilson DJ, Smith JR (2014). Use of intravitreal rituximab for treatment of vitreoretinal lymphoma. Br J Ophthalmol.

[4] Conway MD, Jermak CM, Peyman GA, Swanson HT, Blake DA (2008). Buffering capacity of bovine vitreous. Retina.

[5] Sørensen SPL (1909). Enzyme studies II. The measurement and meaning of hydrogen ion concentration in enzymatic processes. Biochem Zeitschrift.

[6] Reddi BA (2013). Why is saline so acidic (and does it really matter?). Int J Med Sci.

[7] Papadopoulos N, Martin J, Ruan Q, Rafique A, Rosconi MP, Shi E, Pyles EA, Yancopoulos GD, Stahl N, Wiegand SJ (2012). Binding and neutralization of vascular endothelial growth factor (VEGF) and related ligands by VEGF Trap, ranibizumab and bevacizumab. Angiogenesis.

[8] Baino F (2011). Towards an ideal biomaterial for vitreous replacement: historical overview and future trends. Acta Biomater.

[9] Le Goff MM, Bishop PM (2008). Adult vitreous structure and postnatal changes. Eye.

[10] Kokavec J, Min SH, Tan MH, Gilhotra JS, Newland HS, Durkin SR, Grigg J, Casson RJ (2016). Biochemical analysis of the living human vitreous. Clin Exp Ophthalmol.

[11] Shawer M, Coffey MJ, Phillips E. Vitreous humor buffering capacity of rabbi, bovine, and porcine. Invest Ophthalmol Vis Sci ARVO. 2012. 463/D1140. http://iovs.arvojournals.org/article.aspx?articleid=2349281.

[12] Lorget F, Schuetz C, Authier S, Carrier M, Parenteau A, Daughetyl A et al. Characterization of the pH and temperature in the rabbit vitreous: key parameters for the development of long acting delivery of drugs to the posterior segment of the eye. Invest Ophthalmol Vis Sci ARVO 2015. 146/C0001. http://iovs.arvojournals.org/article.aspx?articleid=2331163&resultClick=1.

[13] de Oliveira Dias JR, Badaró E, Novais EA, Colicchio D, Chiarantin GM, Matioli MM, Verna C, Penha FM, Barros NM, Meyer CH, Farah ME, Rodrigues EB (2014). Preclinical investigations of intravitreal ziv-aflibercept. Ophthalmic Surg Lasers Imaging Retina.

[14] Iguchi Y, Asami T, Ueno S, Ushida H, Maruko R, Oiwa K, Terasaki H (2014). Changes in vitreous temperature during intravitreal surgery. Invest Ophthalmol Vis Sci.

[15] Moreno MR, Tabitha TS, Nirmal J, Radhakrishnan K, Yee CH, Lim S, Venkatraman S, Agrawal R (2016). Study of stability and biophysical characterization of ranibizumab and aflibercept. Eur J Pharm Biopharm.

[16] Ranjbar M, Brinkmann MP, Zapf D, Miura Y, Rudolf M, Grisanti S (2016). Fc Receptor inhibition reduces susceptibility to oxidative stress in human RPE cells treated with bevacizumab, but not aflibercept. Cell Physiol Biochem.

