# Correction to: pH of anti-VEGF agents in the human vitreous: low impact of very different formulations

**DOI:** 10.1186/s40942-017-0097-4

**Published:** 2017-10-16

**Authors:** Bianka Sobolewska, Peter Heiduschka, Karl‑Ulrich Bartz‑Schmidt, Focke Ziemssen

**Affiliations:** 1 Center for Ophthalmology, Eberhard-Karl University Tübingen, Elfriede‑Aulhorn‑Straße 7, 72076 Tübingen, Germany; 2 Department of Ophthalmology, University of Münster Medical Center, Münster, Germany

## Correction to: Int J Retin Vitr (2017) 3:22 DOI 10.1186/s40942-017-0075-x

After the publication of this article [1], we were made aware that the osmolarity of aflibercept (Eylea) was incorrect and should have been 286 mOsm and not 1000 mOsm. The correct version of Table 1 is shown in this correction (Table 1).

**Table 1:** The formulations and pH values of anti-VEGF agents

Drug	Concentration (osmolarity)	Dose in the vitreous (4 ml)/dose in 0.002 ml	Formulation	Measured pH	95% CI
Ranibizumab (Lucentis)	10 mg/ml (289 mOsm)	0.5 mg/0.02 mg	10 mM histidine-HCl, 10% α,α-trehalose dihydrate, 0.01% polysorbate 20	5.32	5.0–5.63
Bevacizumab (Avastin)	25 mg/ml (182 mOsm)	1.25 mg/0.05 mg	42 mM NaH_2_PO_4_·H_2_O, 8.45 mM Na_2_HPO_4_, 6% α,α-trehalose dihydrate, 0.04% polysorbate 20	5.91	5.63–6.19
Aflibercept (Eylea)	40 mg/ml (286 mOsm)	2 mg/0.08 mg	10 mM Na_3_PO_4_, 40 mM NaCl, 5% sucrose, 0.03% polysorbate 20	6.05	5.78–6.31
Ziv-aflibercept (Zaltrap)	25 mg/ml (1000 mOsm)	1.25 mg/0.05 mg	100 mM NaCl, 5 mM Na citrate, 5 mM Na_3_PO_4_, 20% sucrose, 0.1% polysorbate 20	6.1	6.05–6.15
Rituximab (Rituxan)	10 mg/ml	1 mg/0.02 mg	154 mM NaCl, 25 mM Na citrate·2 H_2_O, 0.07% polysorbate 80	6.29	5.97–6.61
